# Anxiety Levels Predict Bone Mineral Density in Postmenopausal Women Undergoing Oral Bisphosphonates: A Two-Year Follow-Up

**DOI:** 10.3390/ijerph18158144

**Published:** 2021-07-31

**Authors:** Gabriella Martino, Federica Bellone, Carmelo M. Vicario, Agostino Gaudio, Andrea Caputo, Francesco Corica, Giovanni Squadrito, Peter Schwarz, Nunziata Morabito, Antonino Catalano

**Affiliations:** 1 Department of Clinical and Experimental Medicine, University Hospital of Messina, 98122 Messina, Italy; martinog@unime.it (G.M.); fbellone@unime.it (F.B.); coricaf@unime.it (F.C.); gsquadrito@unime.it (G.S.); nmorabito@unime.it (N.M.); 2 Department of Cognitive Sciences, Psychology, Education and Cultural Studies, University of Messina, 98121 Messina, Italy; cvicario@unime.it; 3 Department of Clinical and Experimental Medicine, University Hospital of Catania, 95123 Catania, Italy; agostino.gaudio@gmail.com; 4 Department of Dynamic and Clinical Psychology and Health Studies, Sapienza University of Rome, 00185 Rome, Italy; andrea.caputo@uniroma1.it; 5 Department of Endocrinology, Research Centre for Ageing and Osteoporosis, Rigshospitalet-Glostrup Hospital, 2100 Copenhagen, Denmark; peter.schwarz@regionh.dk

**Keywords:** clinical psychology, anxiety, adherence, osteoporosis, bone mineral density, postmenopausal women, bisphosphonates, alendronate, risedronate, fracture risk

## Abstract

Clinical psychological factors may predict medical diseases. Anxiety level has been associated with osteoporosis, but its role on bone mineral density (BMD) change is still unknown. This study aimed to investigate the association between anxiety levels and both adherence and treatment response to oral bisphosphonates (BPs) in postmenopausal osteoporosis. BMD and anxiety levels were evaluated trough dual-energy X-ray absorptiometry and the Hamilton Anxiety Rating Scale (HAM-A), respectively. Participants received weekly medication with alendronate or risedronate and were grouped according to the HAM-A scores into tertiles (HAM-A 3 > HAM-A 2 > HAM-A 1). After 24 months, BMD changes were different among the HAM-A tertiles. The median lumbar BMD change was significantly greater in both the HAM-A 2 and HAM-A 3 in comparison with the HAM-A 1. The same trend was observed for femoral BMD change. Adherence to BPs was >75% in 68% of patients in the HAM-A 1, 79% of patients in the HAM-A 2, and 89% of patients in the HAM-A 3 (*p* = 0.0014). After correcting for age, body mass index, depressive symptoms, and the 10-yr. probability of osteoporotic fractures, anxiety levels independently predicted lumbar BMD change (β = 0.3417, SE 0.145, *p* = 0.02). In conclusion, women with higher anxiety levels reported greater BMD improvement, highlighting that anxiety was associated with adherence and response to osteoporosis medical treatment, although further research on this topic is needed.

## 1. Introduction

There is growing interest regarding the crucial role of clinical psychological factors in chronic conditions [1,2,3,4,5]. It is well known that psychological features may help predict medical diseases, referring to both personality traits and mood states (e.g., alexithymia, depression, anxiety) as well as conscious and unconscious strategies (e.g., coping, defense mechanisms) [6,7,8,9,10,11,12,13,14]. On one hand, chronic conditions and their linked outcomes could lead to psychological symptoms, compromising the patients’ health-related quality of life (HR-QoL) [15,16]. On the other hand, psychological aspects may elicit emotional distress, suffering, and the impairment of people’s behavior, as well as medical illness management [17]. Several bodies of evidence support the main relevance of both compliance and adherence aimed at the adequate management of disease, in order to avoid concerns and outcomes that could predict morbidity and mortality independently of numerous confounders. The term adherence captures both compliance and persistence in therapy [18,19,20]. Particularly, adherence is considered as the extent to which patients adequately follow the prescribed treatment, through an active role characterized by a basic therapeutic alliance between the patient and physician. With reference to chronic diseases, adherence to long-term treatments, in developed countries, reaches about 50% one year after the start of therapy, with greater percentages in higher socioeconomic groups [18,19,20,21]. Besides, poor adherence has been associated with higher hospitalization rates, increased morbidity, and mortality. Furthermore, lower adherence could lead to worse perceptions of HR-QoL and to increased health care costs [21,22]. Among chronic diseases, osteoporosis and related fragility fractures are one of the most common cause of pain, disability, and the loss of independence, due to their serious outcomes which significantly impair life expectancy [23,24,25,26]. Particularly, in Europe, the fragility fracture burden is greater than that of many other chronic diseases, representing the fourth cause of total disability after ischemic disease, dementia, and lung cancer, despite other serious medical conditions [23]. Several studies explored the association between clinical psychological factors and fracture risk, suggesting a pathophysiological mechanism underling these two strictly related pathologies [27,28,29,30,31,32,33,34,35]. Kelly et al. analyzed the impact of psychological distress, clinical implication, and treatment interaction, even suggesting the relevance of individualized approaches [9]. Moreover, recent studies explored the significant role of vitamin D and its association with anxious symptoms in postmenopausal women, as well as the predictive role of anxiety in determining a higher fracture risk [35,36,37,38]. Due to several existing therapies [39,40,41,42], which have demonstrated specific efficacy in the treatment of osteoporosis by reducing fracture risk, it could be interesting to investigate, in postmenopausal women, the potential role of psychological features in determining and influencing the osteoporosis treatment response. As well, among several psychological features, anxiety seems to represent a meaningful vulnerability factor, since in chronic conditions individuals fear for the progressive loss of bodily intactness and are faced with managing the unexpected [2,6,8,13]. The aim of this study was to longitudinally investigate the association between anxiety levels and both adherence and treatment response to oral bisphosphonates (BPs).

## 2. Materials and Methods

### 2.1. Participants

This study considered 192 Caucasian postmenopausal women recruited at the Outpatients Clinic for the Prevention and Treatment of Osteoporosis, Department of Clinical and Experimental Medicine, University Hospital of Messina, Italy [35]. Of these women, 128 (median age 68 year, range 35 to 85 year), who had been enrolled between January 2017 and April 2017, received weekly oral antiresorptive medication (i.e., alendronate 70 mg or risedronate 35 mg). They entered this study and followed-up for 2 years. As previously reported [35], we selected women with no current or previous history of neurological or psychopathological disorders, according to DSM-V criteria [43]. We also excluded current or previous physical disorders, such as moderate-to-severe kidney or liver failure, heart failure with a New York Heart Association (NYHA) class ≥ 2, moderate or severe respiratory failure, cancer, malabsorption, endocrine disorders of the thyroid, parathyroid, or adrenal glands, and both psychotropic drugs and active bone agents assumption. The repletion of vitamin D was guaranteed with oral cholecalciferol at a dosage of 25,000 IU every two weeks. The study was carried out in accordance with the ethical standards of the Institutional Ethical Committee, University Hospital “Gaetano Martino”, University of Messina, Italy. Written informed consent was obtained from all the participants. The patients were evaluated by clinical psychologists in collaboration with physicians. All interventions were conducted according to the clinical standard assessment.

### 2.2. Clinical Psychological and Physical Evaluation

At baseline, anxiety levels were evaluated through both a gold standard clinical psychological interview and the Hamilton Anxiety Rating Scale (HAM-A) self-administration, which measured the entirety of anxiety symptoms, based on 14 items referring to psychological and somatic symptoms, including anxiety, tension, fear, insomnia, intellectual impairment, depression, somatic symptoms, sensory, cardiovascular, respiratory, gastrointestinal, genitourinary, autonomic, and observed behavior in the interview. Each item was scored on a Likert scale from 0 (not present) to 4 (severe) [44]. The clinical psychological evaluation also aimed to detect depressive symptoms through the self-administration of the Beck Depression Inventory-second edition (BDI-II), composed of 21 items scored on a Likert scale from 0 (not present) to 4 (severe) [45]. The recruited subjects were divided into tertiles according to their anxiety levels (HAM-A 1 < HAM-A 2 < HAM-A 3). At baseline, FRAX^®^, a computer-based algorithm (http://www.shef.ac.uk/FRAX, accessed on the time of recruitment) was used to estimate both the 10-yr. probability of major osteoporotic and hip fractures, depending on age, body mass index (BMI), prior fragility fracture, parental history of hip fracture, current smoking, alcohol consumption, rheumatoid arthritis, glucocorticoids, and other causes of secondary osteoporosis, as previously reported [46]. A dorsal and lumbar spine X-ray served to locate vertebral fractures when the vertebral body had at least a 20% height reduction in the anterior, middle, or posterior height compared with the same or adjacent vertebra [47]. At baseline and at the end of the study, bone mineral density (BMD) was assessed through the gold-standard dual-energy X-ray absorptiometry (DXA) in antero-posterior projection at the lumbar spine (L1–L4) and femoral neck. Particularly, the DXA densitometer (Hologic Discovery Wi) with a coefficient of variation of 0.5% was calibrated daily, as suggested by the manufacturer’s instruction. Finally, adherence to antiresorptive treatment was investigated through a clinical interview. As well, the percentage of patients who presented an adherence > 75% was evaluated for every identified HAM-A tertile.

### 2.3. Statistical Analysis

Statistical analysis was conducted using MedCalc software (version 10.2.0.0; MedCalc Software Ltd, Mariakerke, Belgium). The normal distribution of values was verified with the Kolmogorov-Smirnov test. The Mann-Whitney test and the Wilcoxon test were used as appropriate. The χ^2^ test was performed to calculate differences in the proportion of the categorical variables. Spearman’s correlation coefficient was used to detect the degree of association between two variables. Multiple regression analysis was applied to analyze the relationship between a dependent variable and one or more independent variables. The values of *p* < 0.05 were considered to indicate statistical significance.

## 3. Results

**Figure 1:**
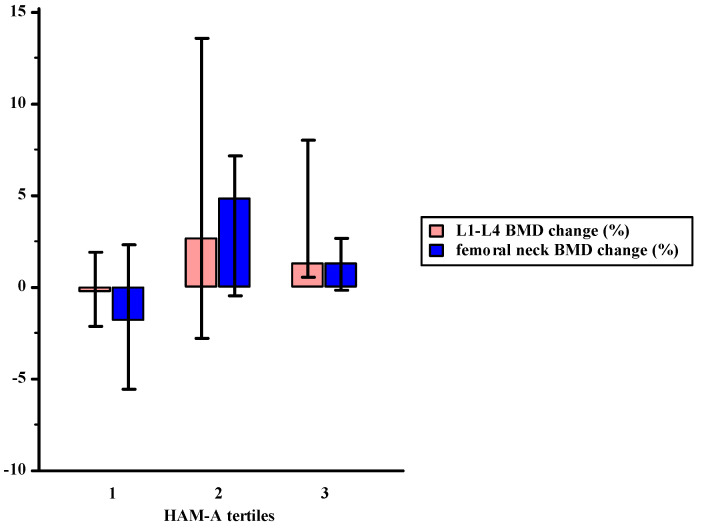
The 24-month BMD change in postmenopausal women treated with weekly alendronate or risedronate, according to tertiles of anxiety levels (HAM-A 1 < HAM-A 2 < HAM-A 3).

A final total of 128 subjects entered the study. Table 1 shows the main clinical characteristics of the recruited postmenopausal women distinguished in tertiles according to anxiety levels. A large number of the patients showed a high education level and more than half were pensioners. The median estimated 10-yr. probability of fractures was 20% and 3.9%, with regard to major osteoporotic and hip fractures, respectively. The median HAM-A score was 28.5, while the median HAM-A somatic and psychic symptom scores were 12 and 16, respectively. As expected, anxiety levels were significantly different among the tertiles, with higher anxious symptoms in the HAM-A 3, in comparison with the HAM-A 2 and HAM-A 1. Educational level was significantly different among the HAM-A tertiles (*p* < 0.001); a significantly higher education level, such as a Bachelors’ degree, PhD, or specialization, was found in the HAM-A 3 tertile in comparison with the HAM-A 1 and HAM-A 2 tertiles (48% vs. 19% and 25%, respectively). Women within the HAM-A 3 tertile, characterized by higher anxiety levels, showed a higher 10-yr. probability of major osteoporotic fractures, according to FRAX, in comparison with participants with lower anxiety symptoms in the HAM-A 1 and HAM-A 2 (25 (21–27.3) vs. 19 (16–22) and vs. 18 (9.6–21.7), *p* < 0.05 and *p* < 0.01, respectively). At baseline, BMD at lumbar spine was significantly lower in the HAM-A 3 (0.75 (0.65–0.83) g/cm²) in comparison with the HAM-A 1 (0.8 (0.74–0.89)), *p* = 0.02, while no significant difference was found in comparison with the HAM-A 2 (0.81 (0.73–0.89)). On the other hand, BMD at femoral neck was not significantly different in the HAM-A tertiles (0.63 (0.58–0.68) g/cm^2^, HAM-A 3; 0.63 (0.57–0.70) g/cm^2^, HAM-A 2; 0.64 (0.59–0.69) g/cm^2^, HAM-A 1). Lumbar and femoral BMD changes at 24 months were significantly different among the HAM-A tertiles. Specifically, the median lumbar BMD change was 1.7% in the HAM-A 3, 5% in the HAM-A 2, and 0.2% in the HAM-A 1, resulting in a significant difference between the HAM-A 1 vs. the HAM-A 2 (*p* = 0.04) and HAM-A 3 (*p* = 0.04) (Figure 1). On the other hand, the median femoral BMD change was 1.3% in the HAM-A 3, 4.8% in the HAM-A 2, and −1.8% in the HAM-A 1, highlighting a significant difference between the HAM-A 1 vs. the HAM-A 2 (*p* = 0.04) (Figure 1).

**Figure 2:**
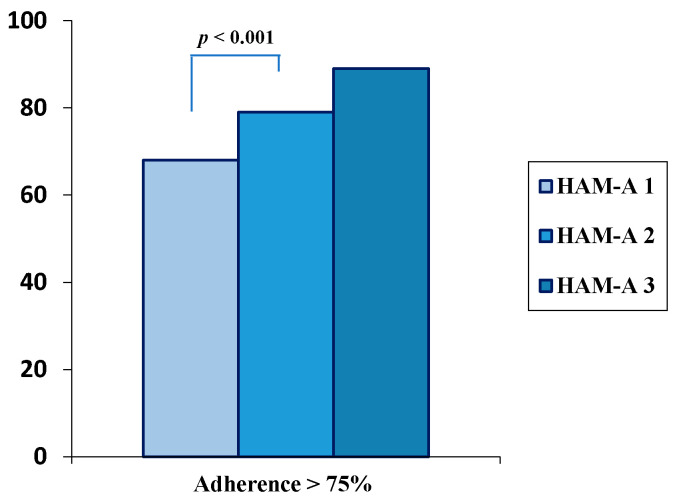
Adherence to weekly alendronate or risedronate in postmenopausal women according to tertiles of anxiety levels (HAM-A 1 < HAM-A 2 < HAM-A 3).

With specific regard to adherence versus alendronate or risedronate, it was >75% in 68% of patients with lower anxiety levels (HAM-A 1), in 79% of patients with middle anxiety levels (HAM-A 2), and in 89% of patients with higher anxiety levels (HAM-A 3) (*p* = 0.0014) (Figure 2). Conversely, no significant difference was found with reference to adherence versus vitamin D, since adherence was >75% in 88% of patients in the HAM-A 1, in 94% of patients in the HAM-A 2, and in 95% of patients in the HAM-A 3 (*p* > 0.05). During the follow-up, neither treatment-related adverse events nor incidents of diseases were reported in the study sample. The multiple regression analysis, selecting lumbar BMD change as the dependent variable and correcting for age, BMI, and the 10-yr. probability of osteoporotic fractures, showed that anxiety levels were independent predictors of lumbar BMD changes at 24 months (β = 0.3417, SE 0.145, *p* = 0.02).

## 4. Discussion

This is the first longitudinal research study aiming to explore the relationship between anxiety levels and bone mineral density (BMD) change after treatment with oral bisphosphonates (BPs), and to evaluate the association between anxiety levels and medication adherence in postmenopausal women followed-up for osteoporosis. In the current study, postmenopausal women assessed for bone health were recruited and evaluated to detect clinical psychological features in association with BMD. Mainly, at baseline, due to different anxiety levels, participants were divided into HAM-A tertiles showing a significant association between higher anxiety levels and lower BMD. Hereby, patients were followed-up for 24 months, receiving weekly oral alendronate or risedronate as an anti-osteoporotic medical treatment, according to good clinical practice [48]. At the end of the follow-up, significant differences were found in BMD changes and treatment adherence among the HAM-A tertiles.

The association between BMD and anxiety levels has been already reported in previous studies [33,34,35], indicating the possible existence of common immunological and endocrinological pathogenetic mechanisms underlying both anxiety and osteoporosis [49,50,51,52,53,54,55]. It has been highlighted that anxious subjects showed increased inflammatory markers such as CRP, and higher pro-inflammatory cytokines as TNFα, IL-1, IL-6, and IL-17, which induce bone resorption, triggering osteoclast function via the RANK-L pathway [49,51]. Moreover, due to the hypothalamic-pituitary-adrenocortical axis stimulation, anxious patients showed higher plasmatic cortisol levels, which may contribute to bone loss through enhancing bone resorption and decreasing bone formation [51,52]. It is also true that oxidative stress may play a relevant role in the pathophysiology of both anxiety and osteoporosis [53,54,55]. In the present longitudinal study, patients with higher anxiety levels (the HAM-A 2 and HAM-A 3 tertiles) reported a significant improvement of BMD, in comparison with those with lower anxious symptoms (the HAM-A 1 tertile). This appears partially in contrast with our previous findings, which pointed out the negative impact of anxiety on bone health. On one hand, the BMD improvement could be explained through the protective effect of the oral weekly anti-osteoporotic treatment, prescribed throughout the 24 months follow-up. Mainly, the BMD improvement along with the adherence evaluation highlighted that only women who persistently assumed the prescribed medical treatment during the follow-up obtained its relative effect on BMD improvement. Conversely, the lack of BMD enhancement has been found in association with lower anxiety levels and poorer adherence to treatment, with reference to women who did not regularly assumed oral BPs. Some studies reported a large number of women do not generally take osteoporotic medication appropriately, decreasing the benefits of the treatment. Particularly, the prevalence of medication adherence has been reported to range from 12.9 to 95.4%, and it has been highlighted that even up to 30% of patients do not start oral BP treatment after prescription [56,57,58,59,60,61,62]. Misconceptions about the risk of the rare BP-related adverse events such as osteonecrosis of the jaw, which remains a rare event in patients treated for osteoporosis, may even facilitate a treatment gap [63,64,65,66]. Hiligsmann et al. showed that the one year BP persistence rate ranges from 16 to 60%, suggesting 40% of patients discontinue treatment within the first 12 months [18]. Several reasons for poor adherence are described by the World Health Organization through five main categories, including patient-related, therapy-related, condition-related, health system, and socio-economic factors [19]. It is also known that the main determinants of poor adherence and treatment discontinuation could be represented by polypharmacy, prevalent gastro-intestinal diseases, low education level, misconceptions about bone fragility, the lack of perceived treatment benefit, iatrogenic side effects, low-income level, and a lack of medical insurance coverage [67]. Significantly, in the present study, participants with better compliance and adherence, together with higher anxiety levels, showed healthier BMD change at the end of the follow-up. This could be due in part to the patients’ possible worry about fractures, which could have encouraged them to adequately assume the prescribed anti-osteoporotic medication [68,69,70]. Additionally, education level has been observed to differ across the HAM-A tertiles; it was higher in women who experienced higher anxiety. In this regard, it could be speculated that higher education levels could have supported compliance and adherence in more anxious women, with a deeper consciousness of their bone metabolic disease and the need for long-term medical treatment. Indeed, previous studies on chronic conditions showed that minimizing the role of illness and lowering anxiety and painful feelings made patients less likely to enact concern for their health and consequent self-restoration strategies, thus negatively affecting treatment adherence [71,72,73]. Similarly, we assume that a lower education level, found in less anxious women, could have negatively influenced compliance, adherence, and illness consciousness, leading to a higher percentage of treatment discontinuation and consequent poorer BMD improvements. Nevertheless, all the recruited women showed a higher adherence, in comparison with the mean Italian data, reflecting an adherence of 60% versus anti-osteoporotic treatment, and suggesting the positive role of a specialized outpatient clinic for the care and management of osteoporosis [74,75]. Although we did not detect side effects due to the oral BP treatment (e.g., gastrointestinal symptoms), we could argue that the rare inadequate BPs assumption could be due to the uncomfortable administration, for which patients were instructed to take BP once a week with a glass of water, avoiding food and drinks for 30 min after morning administration and standing upright for at least 30 min.

It could be speculated that vitamin D supplementation was prescribed in addition to BP and contributed to the BMD changes at the end of the follow-up. In this regard, it should be considered that alendronate and risedronate are recommended for the treatment of osteoporosis based on randomized, placebo-controlled trials, in which vitamin D supplementation was mandatory [76,77,78,79,80,81,82]. Furthermore, the repletion of vitamin D in postmenopausal women suffering from osteoporosis, promoted the osteoporosis treatment response for both BMD changes and anti-fracture efficacy [83,84,85,86,87].

In the current study, unlike adherence to BPs, adherence to vitamin D was similar among the HAM-A tertiles, probably due to the bimonthly vitamin D schedule, the lower probability of adverse events, and the general propensity of its use, as it is considered a panacea for several medical illnesses [88,89,90,91].

We acknowledge that this research study has some limitations. The small sample size and the female sample do not allow us to extend results to the general population under treatment for osteoporosis. The two years’ follow-up provides the evaluation on the interrelationship between clinical psychological factors and BMD, but not with osteoporotic fractures. Moreover, all participants were referred to a single University Outpatients Clinic which represents a possible selection bias; however, this could also be considered a strength due to both the homogeneous clinical psychological interview and administration, as well as to the medical management.

## 5. Conclusions

The present study focused on the association between anxiety levels and treatment response to oral BPs in postmenopausal women with osteoporosis. At the end of the two years’ follow-up, women with higher anxiety levels reported greater BMD improvement, underlying the crucial role of anxiety as a predictor of adherence and response to anti-osteoporotic oral treatment. We consider that patient education and counselling, as well as monitoring and supervision, are required to improve adherence and bone health [62,74]. Since anxiety levels are associated with adherence, and lower anxiety levels are encountered in women with lower adherence, we suggest a personalized clinical psychological and physical approach may promote adherence and drug benefits. Further studies are needed to explore the long-term BMD changes, fracture risk, and psychological features in postmenopausal women undergoing oral BPs treatment.

## Figures and Tables

**Table 1:** Main clinical characteristics of recruited postmenopausal women receiving prescription of weekly alendronate or risedronate as anti-osteoporotic medical treatment.

	Total (*n* = 128)	HAM-A 1 (*n* = 58)	HAM-A 2 (*n* = 32)	HAM-A 3 (*n* = 38)
**Risk factors for osteoporosis**				
Age (year)	68 (67–70)	67 (66–71)	68 (63–70.7)	69 (64–73.6)
Age at menopause (year)	49.5 (47.8–50)	50 (47.2–51.3)	50 (45–52)	47 (44–49.3)
Time since menopause (year)	20 (17–23)	17 (16–24.1)	19 (16–23)	24 (20–27.3)
BMI (kg/m^2^)	24.4 (23.2–25.3)	25 (22.3–26.6)	24.7 (22–31)	23.8 (22.6–25)
Previous fracture (*n* (%))	112 (87.5)	52 (89)	26 (81)	34 (89)
Parent fractured hip (*n* (%))	58 (45.3)	26 (44)	14 (43)	18 (47.3)
Current smoking (*n* (%))	22 (17.1)	6 (10.3)	6 (18.7)	10 (26.3)
Glucocorticoids (*n* (%))	5 (4.68)	2 (3.4)	0	3 (5.2)
Rheumatoid arthritis (*n* (%))	2 (1.56)	0	2 (6.25)	0
Secondary osteoporosis (*n* (%))	40 (31.2)	18 (31)	12 (37.5)	10 (26.3)
Alcohol ≥ 3units/day (*n* (%))	0	0	0	0
**Education**				
Primary school (*n* (%))	23 (18)	14 (24)	6 (19)	3 (8)
Secondary school (*n* (%))	25 (19)	10 (17)	8 (25)	7 (18)
High school (*n* (%))	43 (34)	23 (40)	10 (31)	10 (26)
Bachelors’ degree (*n* (%))	20 (16)	8 (14)	3 (9)	9 (24)
PhD or specialization (*n* (%))	17 (13)	3 (5)	5 (16)	9 (24)
Employment status				
Housewife (*n* (%))	23 (18)	12 (21)	6 (19)	5 (13)
Full-time (*n* (%))	18 (14)	10 (17)	4 (12)	4 (11)
Unemployed (*n* (%))	10 (8)	7 (12)	2 (6)	1 (3)
Pensioner (*n* (%))	77 (60)	29 (50)	20 (63)	28 (73)
**Ten years’ probability of fractures**				
Major osteoporotic fractures (median (IQR))	20 (18.5–22.9)	19 (16–22)	18 (9.6–21.7)	25 (21–27.3) *#
Hip fracture (median (IQR))	3.9 (3–5.4)	3.3 (2.7–6)	3.5 (2.6–6)	5.2 (3.7–9)
**Anxiety levels**				
HAM-A score (median (IQR))	28.5 (26–30)	24 (21–25)	30 (29–31) *	33 (32–35.3) *#
HAM-A somatic symptom score (median (IQR))	12 (11–13)	10 (9–11)	13 (11.2–14) *	16 (14.6–16) *#
HAM-A psychic symptom score (median (IQR))	16 (16–17)	14 (13–14)	17 (16.2–17.8) *	20 (18.6–21) *#
**Depression severity**				
**BDI-II score [median (IQR)]**	7 (6.5–7.4)	5 (4–6)	7 (6–8) *	9 (7–10) *#

HAM-A= Hamilton Anxiety Scale; BDI-II= Beck Depression Inventory-second edition. * *p* < 0.01 vs. HAM-A1, # *p* < 0.05 vs. HAM-A2.

## Data Availability

The data presented in this study are available on request from the corresponding author. The data are not publicly available due to privacy.

## Abbreviations

BDI-II = Beck Depression Inventory-Second Edition
BMD = Bone Mineral Density
BMI = Body Mass Index
BPs = Bisphosphonates
CRP = C-reactive Protein
DSM-V = Diagnostic and Statistical Manual of Mental Disorders, Fifth Edition
DXA = Dual-Energy X-Ray Absorptiometry
FRAX = Fracture Risk Assessment Tool
HAM-A = Hamilton Anxiety Rating Scale
HR-Qol = Health Related Quality of Life
IL = Interleukin
IQR = Inter Quartile Range
IU = International Unit
NYHA = New York Heart Association
RANK-L = Receptor Activator of Nuclear Factor Kappa-Β Ligand
TNF = Tumor necrosis factor
